# A monolithic InP/SOI platform for integrated photonics

**DOI:** 10.1038/s41377-021-00636-0

**Published:** 2021-09-26

**Authors:** Zhao Yan, Yu Han, Liying Lin, Ying Xue, Chao Ma, Wai Kit Ng, Kam Sing Wong, Kei May Lau

**Affiliations:** 1 Department of Electronic and Computer Engineering, Hong Kong University of Science and Technology, Clear Water Bay, Kowloon, Hong Kong China; 2 Department of Physics and William Mong Institute of Nano Science and Technology, Hong Kong University of Science and Technology, Clear Water Bay, Kowloon, Hong Kong China

**Keywords:** Silicon photonics, Nanowires

## Abstract

The deployment of photonic integrated circuits (PICs) necessitates an integration platform that is scalable, high-throughput, cost-effective, and power-efficient. Here we present a monolithic InP on SOI platform to synergize the advantages of two mainstream photonic integration platforms: Si photonics and InP photonics. This monolithic InP/SOI platform is realized through the selective growth of both InP sub-micron wires and large dimension InP membranes on industry-standard (001)-oriented silicon-on-insulator (SOI) wafers. The epitaxial InP is in-plane, dislocation-free, site-controlled, intimately positioned with the Si device layer, and placed right on top of the buried oxide layer to form “InP-on-insulator”. These attributes allow for the realization of various photonic functionalities using the epitaxial InP, with efficient light interfacing between the III–V devices and the Si-based waveguides. We exemplify the potential of this InP/SOI platform for integrated photonics through the demonstration of lasers with different cavity designs including subwavelength wires, square cavities, and micro-disks. Our results here mark a critical step forward towards fully-integrated Si-based PICs.

## Abstract (web summary)

A monolithic InP/SOI platform with dislocation-free, site-controlled and in-plane InP sub-micron wire and large dimension InP membrane array selectively grown on (001) SOI platform.

## Graphical abstract

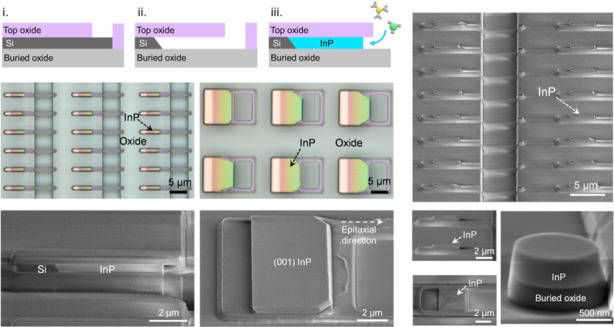

## Introduction

Photonic integrated circuit (PIC) has been the backbone for low-power and high-speed datacom/telecom communication systems^1,2^, and is regarded as the enabling technology for a variety of emerging applications including high performance computing, automobile, quantum communication, optical sensing, etc^3–5^. While current PICs address the “communication bottleneck” through interfacing different chips and systems using light, future PICs are rapidly evolving from this intra-chip optical interconnect towards inter-chip optical interconnect and even close integration with silicon (Si) electronic circuits^6,7^. Commercial deployment of PICs in these myriad applications especially consumer-oriented ones demands an integration platform that is scalable, cost-effective, and power-efficient^8–10^. Currently, indium phosphide (InP) and Si are the two mainstream photonic integration platforms. While InP photonics realizes all optical functionalities, including light emission, transmission, modulation and detection using InP-based alloys, photonic devices and circuits are built on small-sized and expensive InP substrates and produced in low-volume III–V photonic lines^11^. In contrast, Si photonics benefits from the mature CMOS manufacturing capabilities and allows for the processing of Si-based PICs with ultra-high volume and low cost^12,13^. Nevertheless, due to the indirect band structure of the Group IV materials, Si photonics often requires the integration of III–V lasers for efficient light generation^14^. Integrating III–V materials, in particular InP, on Si wafers therefore has been extensively pursued in the past decades to synergize these two photonic platforms^3,13,15,16^. Heterogeneous integration of III–V lasers on Si using bonding or transfer printing has reached the stage of commercialization due to the high quality of bonded III–V materials^12,13^. Monolithic integration of III–V materials on Si wafers via direct hetero-epitaxy produces III–V/Si platforms in a low-cost and scalable manner, and represents the ultimate solution for fully-integrated PICs^9,10,15^. Recently, blanket hetero-epitaxy of III–V thin films on Si substrates produces III–V/Si templates with low defect densities, and quantum dot lasers grown on these templates exhibit impressive performances^10,17,18^. However, the thick buffers (usually a few micrometers) required for defect reduction complicate the light coupling between the III–V lasers grown on top and the Si-based photonic components underneath. In addition, despite the low defect densities (in the order of 10^6^ cm^−2^) obtained in the GaAs/Si templates^17^, the InP/Si templates, where most 1.5 µm band telecom optoelectronic devices are built on, still manifest a high defect density (in the order of 10^8^ cm^−2^)^19^. As an alternative, selective hetero-epitaxy creates a complete suite of dislocation-free III–V crystals on Si, and simultaneously ensures an intimate placement between the epitaxial III–V and the Si substrate^20–31^. However, due to the mandatory defect necking effects, the volume of the selectively grown III–V materials is often very limited, and expanding the material dimension and subsequent realization of electrically driven lasers have been extremely challenging^32^.

Ideally, fully-integrated PIC necessitates a monolithic III–V/Si platform with an intimate positioning of low-defect III–V and Si for efficient light coupling and large dimension III–V materials for flexible device/circuit designs. Both the conventional blanket and selective hetero-epitaxy schemes have yet to offer an ideal solution. Here, we report a monolithic InP/SOI platform for integrated photonics, where both dislocation-free InP sub-micron wires and large dimension InP membranes are selectively grown on (001) SOI wafers and intimately placed with the Si device layer. These InP crystals locate right on top of the buried oxide layer and feature an in-plane configuration with the Si device layer, which results in a unique InP-on-insulator architecture and allows for strong light confinement within the epitaxial InP. As a result, we obtained room temperature and site-controlled laser array from the InP subwavelength wires, and square cavity lasers from as-grown InP crystals. The large dimension InP membranes allow for additional top-down processing and we exemplify this capability through fabricating room temperature micro-disk lasers. More importantly, the InP membranes could potentially serve as templates for the subsequent regrowth of numerous InP-based optoelectronic structures. Our monolithic InP/SOI platform here represents an elegant synergy of the conventional InP and Si photonic platforms and marks an important step towards fully-integrated PICs.

## Results

### InP sub-micron wire and membrane array selectively grown on SOI

**Fig. 1:**
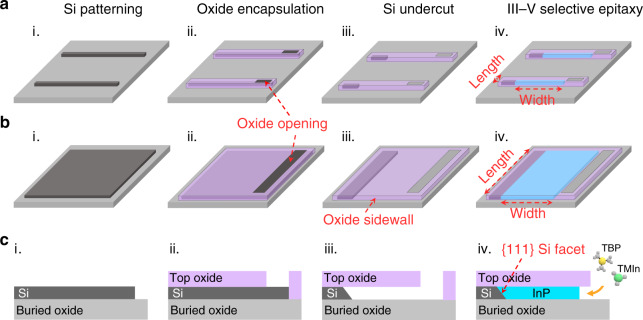
The concept of growing in-plane InP sub-micron wire and membrane array on (001) SOI wafers. **a**, **b** Schematic of selective lateral hetero-epitaxy of InP sub-micron wires and membranes. **c** Cross-sectional schematics of each step. (i) Si device layer patterning. (ii) Oxide encapsulation (oxide deposition and opening). (iii) Anisotropic Si wet etching to form Si undercut. (iv) Selective epitaxy of in-plane InP sub-micron wires and membranes. Definition of InP sub-micron wires and membranes dimensions: the width refers to the dimension along lateral epitaxial direction, while the length denotes the orthogonal dimension of the oxide patterns defined by lithography, as indicated in step iv

Figure 1 schematically depicts the selective area growth of site-controlled and in-plane InP sub-micron wires and membranes on (001)-oriented SOI wafers. We leveraged our previously devised “lateral aspect ratio trapping (ART)” method to enable the epitaxy of InP crystals inside lateral oxide trenches enclosed by the top oxide layer and the buried oxide layer^31^. In our previous works, we have demonstrated the growth of micrometer-scale InP stripes with a width (the dimension along the depth of the oxide trench) up to 3.0 µm on SOI^32^ (refer to Fig. 1). In this article, apart from the epitaxial confinement of the top/bottom layers, we added additional confinements through incorporating the concept of “template assisted selective epitaxy” (TASE) and introducing two lateral oxide sidewalls. In addition, we create ultra-deep lateral oxide trenches with a width larger than 7.0 µm and an aspect ratio larger than 14, and take full advantage of the diffusive characteristics of organometallic precursors in the metal organic chemical vapor deposition (MOCVD) systems. These unique features enable the growth of dislocation-free InP segments with a thickness of 480 nm, a width adjustable from a few hundred nanometers to 7.0 micrometers, and a length tunable from a few hundred nanometers to tens and possibly hundreds of micrometers. Note that III–V grown by TASE initiates III–V nucleation from a tiny Si seed area (down below 100 nm) to promote elastic relaxation and form dislocation-free III–V nanowires and micro-cavities^25,26^. In our lateral ART method, InP nucleates on a larger Si area and dislocations generated from the InP/Si interface are trapped by defect necking effect; both in-plane InP sub-micron wires and large dimension InP membranes could be simultaneously produced on (001) SOI substrates in this manner. As indicated by the schematics in Fig. 1a, b, the length of the epitaxial InP segments hinges on the growth patterns defined by photolithography process; when the length is at sub-micrometer scale, InP sub-micron wires are formed; as the length increases to micrometer scale, the InP sub-micron wires accordingly evolve into InP membranes. The thickness of the epitaxial InP is identical to that of the Si device layer, and the width depends on the undercut of the lateral oxide trench and is further tailored by the growth time. We carefully devised a set of growth parameters to enable the diffusion of the growth precursors into the deep lateral oxide trench and uniform deposition of InP on the exposed {111}-oriented Si surfaces (see methods). In our previous works of InP growth on Si, GaAs nucleation is used as the stress-reducing buffer between InP and Si^23^. In this work we eliminated the GaAs intermediate layer and adopted a two-step growth procedure, consisting of a low temperature InP nucleation layer and a high temperature InP main layer. The thin InP nucleation was grown at 400 °C with a V/III ratio of 1400 and a growth pressure of 50 mbar, which enables the lateral diffusion of growth precursors into the 7.0 µm wide oxide trench and the resultant deposition of uniform InP islands on the Si surface. The main InP growth was performed at 620 ^o^C with a V/III ratio of 20, which results in the coalescence of InP islands into continuous crystals and the expansion of the width of the epitaxial InP. Antiphase boundaries (APB) and threading dislocations (TD), the two most detrimental crystalline defects in III–V hetero-epitaxy on Si^33^, are essentially eliminated. In our design, the formation of APBs is avoided through initiating III–V growth on {111}-oriented Si surface formed by anisotropic wet etching^20,34^. Deposition of InP on {111} Si facets also promotes stress-relaxation via a highly twinned region at the InP/Si interface instead of more detrimental TDs. In addition, the large aspect ratio up to 14 effectively blocks all the TDs and gives rise to TD/APB-free InP sub-micron wires and membranes right on top of the buried oxide layer.

**Fig. 2:**
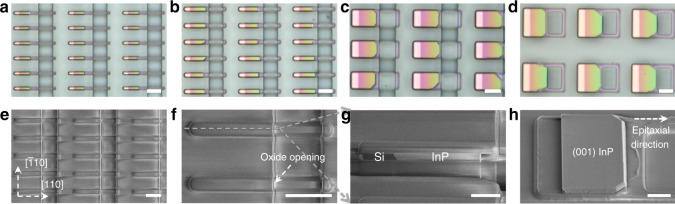
In-plane InP sub-micron wires and membranes selectively grown on (001) SOI. **a**–**d** Optical microscope images of as-grown InP array with different pattern length: 500 nm, 1 µm, 5 µm, and 10 µm, respectively. Scale bar of **a**–**d**, 5 µm. **e**, **f** Top-view SEM image of as-grown sample at 1 µm patterned length area. Scale bar of **e**, **f**, 5 µm. **g** 52° tilted cross-sectional SEM image after focus-ion-beam cutting showing the in-plane InP laterally grown from Si. Scale bar, 2 µm. **h** 52° tilted SEM image of InP membrane grown by 10 µm pattern length after top oxide and Si removal. Scale bar, 2 µm

Figure 2 displays the microscopic and scanning electron microscopic (SEM) photos of the InP sub-micron wires and membranes grown on SOI using the lateral aspect ratio trapping approach. InP sub-micron wire and membrane arrays with different lengths can be formed in a single growth run through designing different growth patterns. Figure 2a, b presents the microscopic images of InP sub-micron wire arrays with a pattern length of 500 nm and 1.0 µm, respectively. The color change in the microscope images results from the slight change of the thickness of the top oxide layer and the InP layer underneath (see Supplementary Section I). Figure 2e, f show the SEM photos of the InP sub-micron wire array imaged from different angles and magnifications; Fig. 2g displays the cross-sectional SEM photo. Guided by the pre-defined oxide patterns, the growth of the InP sub-micron wires started from the etched {111}-oriented Si facets and evolved towards the pattern opening. The width of the InP wire with a pattern length of 500 nm is ~4.0 µm, while that of the InP wire with a pattern length of 1.0 µm is ~6.0 µm. The larger width stems from the larger pattern opening and resultant more diffused growth precursors. In contrast to vertical III–V nanowires in the literature, our InP sub-micron wires feature an in-plane configuration with the Si device layer with pure axial growth. This distinctive feature brings out a few unique advantages. Firstly, the InP sub-micron wire array is grown on (001)-oriented SOI wafers while vertical III–V nanowires are often grown on {111} Si or native III–V substrates; secondly, the in-plane characteristic is more compatible with the planar processing technologies in the CMOS foundries; thirdly, the InP sub-micron wire array exhibits an extremely intimate positioning with the Si device layer, which allows for seamless light interfacing with Si-waveguides; finally, the pure axial growth guided by oxide patterns produces sub-micron wires with different geometries, for example square cross-section in this work, and allows for similar epitaxial flexibilities in conventional planar epitaxial processes^27^. Figure 2c, d displays the microscopic images of InP membrane array with a pattern length of 5 µm and 10 µm, respectively. Similar to the InP sub-micron wires, the epitaxial width of the InP membranes laterally extended to 7 µm and gradually filled up the growth pattern without any visible gaps or voids. Unlike the sub-micron wires where the epitaxial width hinges on the size of the pattern openings, the InP membranes with different pattern lengths exhibit a similar epitaxial width of 7.0 µm due to the sufficiently large pattern openings. The in-plane growth rate of our InP membrane is ~5.0 µm/hr, and this rate can be easily varied through adjusting the amount of the growth precursors. The InP membranes show a multi-faceted growth front with two sloped facets near the lateral oxide sidewalls. This kind of multi-faceted growth front is not uncommon in selective area epitaxy and results from the tendency to minimize the total surface energy during the epitaxy process^35^. We highlight the surface morphology of the InP membrane in Fig. 2h after removal of the top oxide layer. The InP membrane manifests an atomic-flat (001)-orientated top facet and a unique InP-on-insulator characteristic, an architecture similar to InP membranes bonded on SOI wafers. These large-area InP membranes overcome the inherent issue of limited material volume in conventional selective hetero-epitaxy and hold the potential of realizing electrically driven lasers with minimized metal induced absorption loss. The InP membrane-on-insulator can also be fabricated into a variety of III–V micro/nano-structures using conventional top-down processing; examples include nanowires, nano-fins, micro-disks, and micro-rings. In addition, the InP membranes can serve as templates for the regrowth of numerous III–V structures and subsequently enable the realization of various III–V optoelectronic devices. These unique features render the InP-on-insulator an elegant platform for the implementation of photonic functionalities.

### Material characterizations

**Fig. 3:**
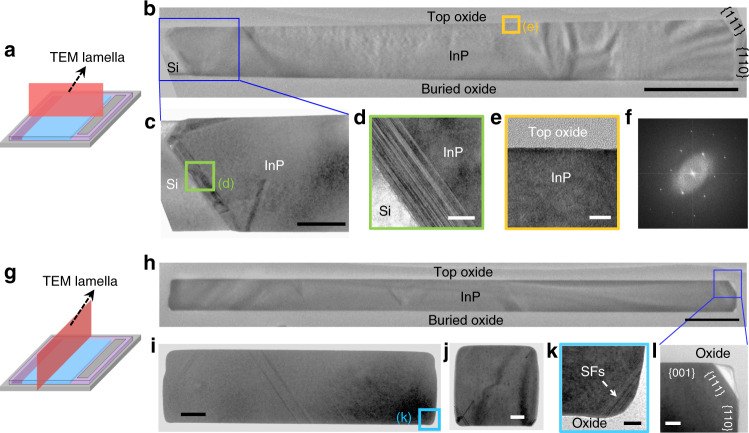
TEM characterization of InP sub-micron wires and membranes. **a** Schematic to illustrate the TEM lamella along epitaxial direction. **b** Global-view TEM image of InP membrane by 10 µm pattern length. Scale bar, 1 µm. **c** TEM image close to Si area. Scale bar, 200 nm. **d** Close-up TEM image at InP/Si interface. Scale bar: 5 nm. **e**, **f** Representative TEM image at main epitaxial area and the corresponding FFT image. Scale bar, 10 nm. **g** Schematic to illustrate the TEM lamella perpendicular to epitaxial direction. TEM image of InP membrane (**h**, **i**) with 10 µm and 2 µm pattern length, and InP sub-micron wire (**j**) with 500 nm pattern length, respectively. Scale bar of **h**–**j**, 1 µm, 200 nm, and 100 nm, respectively. **k** Zoomed-in TEM image of **i** at bottom-right oxide corner. Scale bar, 20 nm. **l** Zoomed-in TEM image of **h** at upper-right corner. Scale bar, 100 nm

We inspected the crystalline quality of the InP sub-micron wires and membranes as well as the defect necking effect of the lateral ART approach through extensive transmission electron microscopy (TEM) characterizations. As indicated by the schematic in Fig. 3a, we first prepared the TEM lamellae along the epitaxial direction using focus-ion-beam (FIB). We investigated TEM specimens of InP segments with different pattern length and observed similar crystalline quality and defect management mechanisms. Figure 3b displays a representative global-view TEM image of an InP membrane with a pattern length of 10 µm. The epitaxial InP is sandwiched between the top and the buried oxide layer without any observable TDs. The growth front of the InP membrane contains a top (111), a middle (110), and a tiny bottom $(\bar 1\bar 11)$ facets, suggesting an identical crystalline structure as the Si device layer. The crystalline structure of the InP follows that of the Si device layer and is confirmed as zinc-blende as evidenced by the zoomed-in TEM photo in Fig. 3e and the Fast-Fourier transform (FFT) diffraction pattern in Fig. 3f. Inspection of the InP/Si hetero-interface reveals that the stress induced by lattice mismatch is relaxed via the formation of a high density of stacking disordered layers (Fig. 3c, d). The majority of these {111}-oriented stacking disorders run parallel to the Si surface and terminate at the top and the buried oxide layers. Benefited from the large aspect ratio of the lateral oxide trenches, any TDs propagating along the {111} planes are blocked by the top oxide layer^33^ (see Supplementary Fig. S3 for details). We also prepared TEM lamellae perpendicular to the epitaxial direction to further evaluate the crystalline quality of the epitaxial InP as indicated by the schematic in Fig. 3g. Figure 3h, i display the cross-sectional TEM photos of InP membrane with a pattern length of 10 µm and 2 µm, respectively, and Fig. 3j presents the cross-sectional TEM photo of InP sub-micron wire with a pattern length of 500 nm. The TEM lamellae are around 2.6 µm, 3.9 µm, and 2.3 µm away from the Si in these three TEM inspections, respectively. As expected, the InP is encapsulated by oxide in all directions and exhibits a dislocation-free characteristic, benefited from the large distance away from Si. However, we do observe two kinds of stacking faults (SFs) inside all specimens: one type inside the InP and the other at the oxide corner. The first type of SFs originates from the InP/Si hetero-interface^20^; unlike SFs in conventional blanket hetero-epitaxy which are accompanied by dislocations at both ends, these SFs extend across the entire epitaxial InP and thus avoid introducing unwanted nonradiative recombination centers inside the InP^23^ (refer to Supplementary Section II for more discussion). The other type of SFs form at some oxide corners and terminate at the oxide sidewall as indicated by the zoomed-in TEM photos in Fig. 3k. Some of the oxide corners with enclosed {111} InP facet forming an air gap (see Fig. 3l) are without formed SFs. These stacking disorders possibly stem from the surface imperfections at the confined oxide corners, which has been widely noted in conventional selective hetero-epitaxy. The density of these two kinds of SFs can be reduced through optimizing the growth parameters of the InP nucleation and fabricating growth patterns with smoother oxide sidewalls.

**Fig. 4:**
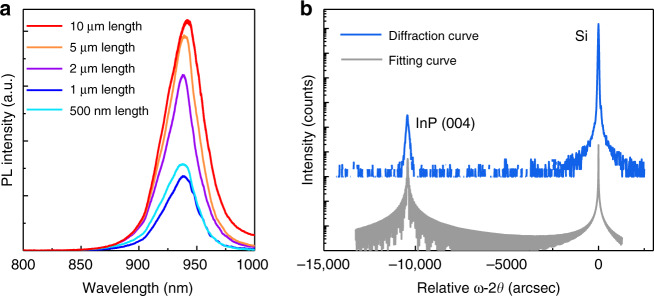
Optical and crystalline characterizations. **a** Room temperature PL spectra of as-grown InP with different pattern length. **b** XRD relative ω-2θ curve of as-grown InP array, and fitting curve of 100% relaxed InP films on Si

We also characterized the InP sub-micron wires and membranes using room temperature micro-photoluminescence (PL) and X-ray diffraction (XRD) measurements. A 514 nm CW laser focused into a 3.5-µm spot diameter was used as the excitation source to measure a single InP segment (see Methods section). Figure 4a plots the room temperature PL spectra of InP wires with a pattern length of 500 nm and 1.0 µm, as well as InP membranes with a pattern length of 2.0 µm, 5.0 µm, and 10.0 µm. The PL peak locates at 925 nm, suggesting a zinc-blende crystal phase. In general, the intensity of the PL emission strengthens as the material volume of epitaxial InP increases. The PL intensity of InP membranes with 5 µm and 10 µm pattern length is comparable because the pumped InP volume in both has the same dimension as the excitation laser spot (see Methods section). We also observed slightly higher PL intensity of the InP sub-micron wires with 500 nm pattern length than the ones with 1 µm pattern length, possibly due to the optical confinement in the nano-scale InP cavity. The linewidth of the PL emission is extracted to be ~28 nm, a value comparable to that of commercial InP wafers^32^. It is worth noting that while a pure zinc-blende phase is observed in the InP membranes, we detected the existence of the wurtzite phase in a small portion of InP sub-micron wires as evidenced by the PL emission at 880 nm (refer to Supplementary Fig. S5 for more discussions). Figure 4b displays the XRD relative ω−2θ scan curve of the as-grown InP segments, revealing sharp peaks of both the epitaxial InP and the Si layer. The full-width at half-maximum (FWHM) of the InP peak is extracted to be as narrow as 90 arcsec benefiting from the elimination of TDs inside the epitaxial InP. XRD fitting to assess the strain of epitaxial InP indicates that the InP is fully relaxed as indicated by the accurate alignment between the measured InP/Si diffraction peaks and fitting peaks in Fig. 4b (refer to Methods section).

### Subwavelength laser arrays and micro-disk lasers on SOI

**Fig. 5:**
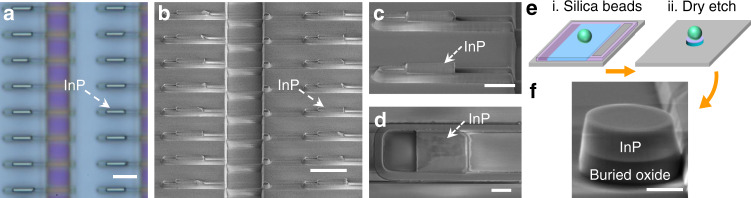
Laser demonstrations using the epitaxial InP sub-micron wires and membranes. **a** Optical microscope image of InP subwavelength wire lasers with 500 nm pattern length after top oxide and Si removal. Scale bar, 5 µm. **b** 52° tilted SEM image of InP subwavelength laser array on SOI by 500 nm pattern length. Scale bar, 5 µm. **c** 52° tilted SEM image of subwavelength lasers. Scale bar, 2 µm. **d** Top-view SEM image of the square cavity laser. Scale bar, 1 µm. **e** Schematic of process flow of micro-disk lasers. **f** 70° tilted SEM image of fabricated micro-disk laser on SOI. Scale bar, 500 nm

The epitaxial sub-micron wire and membrane arrays feature a unique InP-on-insulator characteristic and is an ideal platform for implementing photonic functionalities. To demonstrate the versatility and flexibility of the InP/SOI platform, we fabricated InP subwavelength laser arrays from the wires, and square cavity lasers and micro-disk lasers from the membranes with additional top-down processing. Figure 5a shows a microscope image of InP laser array with a pattern length of 500 nm, and Fig. 5b displays the tilted-view SEM photo. As InP emits ~920 nm and Si is not transparent in this wavelength range, we selectively removed the Si device layer after etching away the top oxide layer (see Methods section). The finalized InP subwavelength wire lasers sit right atop the buried oxide layer and exhibit a Fabry-Perot (FP) cavity with a cavity length of ~3.0 µm and optical feedback from the InP/air interface at both ends (see the zoomed-in SEM photos in Fig. 5c). Conventionally, III–V nanowire lasers are often transferred onto low-index substrates to support strong optical confinement and thus exhibit random orientations and positions. Although nanowire lasers have been demonstrated on native III–V substrates or (111) Si substrates, they often feature a vertical configuration^36^. In sharp contrast, the position and orientation of the subwavelength wire lasers in this work can be precisely controlled via lithography and the lasers exhibit an in-plane configuration and same level with the Si device layer. The large dimension InP membranes right atop the insulator could be defined into a variety of photonic geometries and we illustrate this versatility through fabrication of square cavity and micro-disk lasers. Figure 5d presents the top-view SEM image of a square cavity laser through tailoring the epitaxial width close to the pattern length; the length of the square cavity laser is ~2.0 µm and the side is measured as 2.2 µm. We adopted colloidal lithography to define the micro-disks as illustrated by the schematics in Fig. 5e (refer to Methods section). Figure 5f shows a typical SEM image of the fabricated air-cladded micro-disk lasers on the buried oxide layer with circular cavities and smooth sidewalls; the diameter of the micro-disk laser is measured to be ~1.4 µm.

**Fig. 6:**
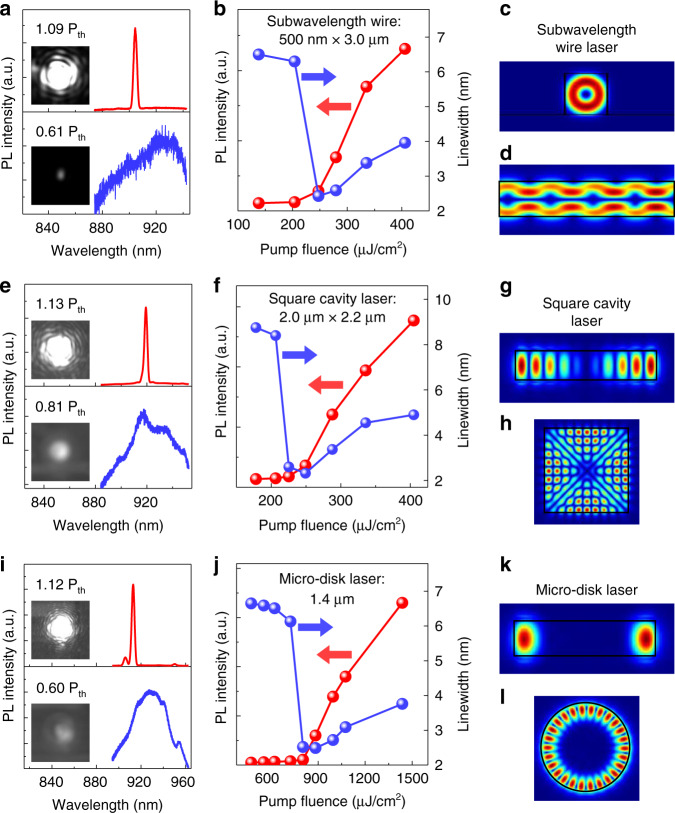
Room temperature lasing of subwavelength wire, square cavity, and micro-disk lasers. **a**, **e**, **i** Room-temperature PL spectra of the subwavelength wire, square cavity, and micro-disk lasers, respectively. Inset shows the corresponding emission images below and above threshold. **b**, **f**, **j** Light–light (L–L) curve and linewidth narrowing of corresponding lasers. Calculated cross-sectional (**c**, **g**, **k**) and top-view (**d**, **h**, **l**) lasing mode profiles of the InP lasers

We probed the optical properties of the subwavelength wire, square cavity, and micro-disk lasers using a home-build PL system, and these lasers can all operate at room temperature under optical excitation. The excitation source was from a 750-nm Ti-Sapphire laser with 100 fs pulse duration and 76 MHz repetition rate (see Methods section). Figure 6a displays the room temperature PL spectra of a subwavelength wire laser with a pattern length of 500 nm and an epitaxial width of 3 µm. At low pump power, we detected a broad PL emission with a peak at ~920 nm; above threshold, a sharp peak at 905 nm stands out from the spontaneous emission and exhibits a linewidth of 2.4 nm. The insets in Fig. 6a display the recorded images of the subwavelength laser below and above threshold, contrasting the faint and uncoherent emission before lasing and the bright and coherent emission after lasing. Figure 6b plots the light–light (L–L) curve and the evolution of the linewidth of the measured wire laser; the clear kink in L–L curve together with the linewidth narrowing further attest to the room temperature lasing behavior. We also performed simulations to study the lasing mode of the wire laser. From calculation of mode reflectivity, the TEM_1,0_ mode exhibits highest end-facet reflectivity (~84% at 905 nm) and thus is most likely to be the lasing mode. Figure 6c, d present the simulated TEM_1,0_ mode from different angles. Figure 6e displays the room temperature emission spectra of a square cavity laser with a length of 2.0 µm, and Fig. 6f plots the evolution of the emission intensity and the linewidth as the pump power strengthens. Similar to the wire laser, we observed a lasing peak at 918 nm, with clear fringe patterns above threshold, obvious kink in the L–L curve and evident linewidth narrowing around threshold. Figure 6g, h displays the cross-sectional and top-view electric field profiles of the whisper-gallery (WG)-like mode of the square cavity; the resonant wavelength of which agrees well with the experimental results. Figure 6i plots the room temperature emission spectra of a micro-disk laser with a diameter of 1.4 µm, and the inset presents the recorded images of the micro-disk laser below and above threshold. In this case, the lasing peak locates at 913 nm, the intensity and linewidth of which is plotted in Fig. 6j. Figure 6k, l plots the cross-sectional and top-view mode profiles of the lasing TE_13,1_ mode with mode locating at the periphery of the micro-disk cavity. Room temperature lasing from these lasers with different types of cavities evidences the excellent quality of the InP subwavelength wires and membranes selectively grown on SOI wafers and demonstrates the flexibility and great potential of this InP/SOI platform for future integrated photonics.

## Discussion

In summary, we presented a monolithic InP/SOI platform with in-plane, site-controlled and dislocation-free InP sub-micron wires and large dimension membranes selectively grown on Si. This InP/SOI platform features a unique InP-on-insulator characteristic and an intimate placement of the epitaxial InP and the Si device layer. We exemplified the potential and versatility of this platform for integrated photonics through the design and demonstration of room temperature subwavelength wire, square cavity, and micro-disk lasers. This is an important step towards fully-integrated PICs as it addresses the critical issues of both the conventional III–V/Si blanket hetero-epitaxy and selective hetero-epitaxy. In conventional blanket hetero-epitaxy, the major roadblocks are the high TD densities of the III–V/Si templates and the weak light coupling between the epitaxial III–V and the Si substrate through the thick III–V buffers. The InP/SOI platform presented here overcomes these blockades through the direct growth of TD-free and buffer-less InP on SOI; our recent simulation studies suggest that this InP/SOI configuration supports efficient light interfacing between the epitaxial InP and the Si-waveguides via both evanescent and butt coupling schemes^21^. In traditional selective hetero-epitaxy, the main obstacle is the limited material volume of the epitaxial III–V and the resultant difficulty in realizing electrically driven lasers. The large dimension InP membranes here could potentially solve this issue. Using the InP membranes on insulator as template, buried hetero-structure including InGaAs quantum wells or InAs quantum dots could be selectively regrown atop the membrane and laser diodes with lateral p-i-n structure could be formed^37^. Through patterning metal contacts at the two ends of the doped membrane away from the guided optical modes, optical absorption loss induced by the metal contacts could be minimized. Efficient light interfacing between the III–V laser and the Si-waveguides could also be simultaneously ensured. In addition, the InP membrane guarantees a large metal contact area with minimal contact resistance. The entire laser structure could also leverage the large dimension InP membrane to avoid the defective InP/Si interface^38^. Based on the targeted device functions, the InP membranes could also serve as templates for the regrowth of a variety of III–V structures for light emission, modulation, and detection^39^. The InP sub-micron wires, formed through the replacement of Si sub-micron fins, are ideal building blocks for subwavelength PICs and could potentially enable the close integration with Si-based nano-electronics. In addition, the attribute of InP-on-insulator resembles InP directly bonded onto oxide layers, and accordingly our InP/SOI platform could benefit from the well-established processing technologies in the III–V heterogeneous integration approach^40^.

## Materials and methods

### InP sub-micron wires and membranes selectively grown on SOI

We used (001)-oriented SOI substrates for the epitaxial growth of InP. The thickness of the Si device layer was thinned from 1.5 ± 0.08 µm down to ~480 nm by a cycled thermal oxidation/wet etching process. The thinned Si device layer was firstly patterned into Si segments with identical width but varied length along [110] direction. Low temperature oxide (LTO) layer was then deposited to maintain the Si segments dimensions, and 5 µm wide oxide opening was etched to partially expose the Si surface for the following Si wet etch. We used 10% diluted KOH solution at room temperature (~25 °C) to selectively etch the Si to desired undercut. This KOH etching condition aims to obtain a high etch selectivity between Si and SiO_2_ and thus create uniform lateral oxide trenches. The etch selectivity is experimentally determined to be around 100:1 for Si and SiO_2_. Before growth, the wafer was cleaned by standard RCA (NH_4_OH:H_2_O_2_:H_2_O = 1:1:5) process and fresh {111} Si facet was created for InP growth according to the following treatment: a brief diluted HF dip to remove native oxide on Si; {111} Si facet etch using 45% KOH solution with isopropanol (IPA) saturation at 70 °C; 10% HCl solution cleaning for 1 min and rinsed with deionized (DI) water. This KOH etching prior to growth was adopted to create a fresh and smooth {111} Si facet to facilitate uniform deposition of III–V nucleation layer on Si. The sample was then immediately loaded into growth chamber. We performed selective epitaxy of InP using an Aixtron closed-couple-showerhead (CCS) MOCVD system with H_2_ as carrier gas. The native oxide on Si was firstly thermally desorbed at 800 °C for 15 min. The InP growth was performed using trimethylindium (TMIn) and tertiarybutylphosphine (TBP) as growth precursors.

### Material characterizations

Micro-PL characterization was performed using a surface-normal pump/detection PL configuration. The pump source was a 514-nm CW laser with 1.25 mW power. The focused laser spot diameter was around 3.5 µm to measure different epitaxial areas. The InP emission was then detected by a thermoelectric cooled InGaAs detector array. There were filters used to block the excitation light from reaching detectors. The XRD characterization was performed using an Empyrean (PANalytical) model for high resolution X-ray diffraction purpose. The X-ray spot was line-shape focused, with a length of 12 mm and a width of ~1.8 mm to measure large-area materials. The XRD fitting was done by a software tool for X-ray diffraction analysis released by the same company.

### Laser fabrication and characterization

The top oxide of subwavelength wire and square cavity lasers was removed by C_4_F_8_/He/H_2_ gas mixture based dry etch. Afterwards, the Si was removed by an additional dry etch step. The micro-disk laser was fabricated using colloidal lithography with 1.5 µm diameter silica beads as initial etching masks. Good circularities were then transferred to the oxide hard mask underneath the silica beads. Afterward, InP disks were formed using inductively coupled-plasma (ICP) etching with top oxide disk as hard mask. Finally, the oxide mask atop was removed by dry etch. The strong adhesion between these InP lasers and the buried oxide layer can endure various processing steps, including ultrasonic processing, dry etch, ALD at 300 °C, etc, without any detachment. The subwavelength wire, square cavity, and micro-disk lasers were characterized using a micro-PL system with a pulsed laser source. The pump power density was calibrated as the power reaching the InP cavity without considering the multiple reflections at the InP/Air and the InP/oxide interfaces. In our previous work where dimension of the excitation laser spot is comparable to the dimension of the laser cavity, we determined the pump power density by directly using pump power divided by the area of pump light^23^. However, in this measurement, the size of the pump light (10 µm diameter) is much larger than the dimension of InP lasers. As a result, we calibrated the pump power density by firstly calculating the power incident on the InP (integration of pumped Gaussian beam intensity at middle InP area), and then divided by the area of each structure of InP lasers to better estimate the power density. The scattered PL signal of the InP lasers was collected by an InGaAs detector from top direction. The detector was thermoelectrically cooled to −10 °C for higher sensitivity. The InP laser emission images below and above threshold were taken by a Si-based CMOS camera.

## Supplementary information

Supplementary Information

## Data Availability

The data that support the findings of this study are available from the corresponding author upon reasonable request.
