# Supplementary material for: A monolithic InP/SOI platform for integrated photonics
Source: Light Sci Appl. 2021 Sep 26;10:200. doi: 10.1038/s41377-021-00636-0 (PMC8473568; doi:10.1038/s41377-021-00636-0)
Supplement: Supplementary file 1 — Supplementary Information [file 41377_2021_636_MOESM1_ESM.pdf]

Supplementary Information for

A monolithic InP/SOI platform for integrated photonics

Zhao Yan [a) 1], Yu Han [a) 1], Liying Lin [1], Ying Xue [1], Chao Ma [2], Wai Kit Ng [2], Kam Sing Wong [2], and Kei May Lau * [1]

[1] Department of Electronic and Computer Engineering, Hong Kong University of Science and Technology, Clear Water Bay, Kowloon, Hong Kong, China

[2] Department of Physics and William Mong Institute of Nano Science and Technology, Hong Kong University of Science and Technology, Clear Water Bay, Kowloon, Hong Kong, China

[a)] Zhao Yan and Yu Han contributed equally to this work

* Tel: (852)23587049, Fax: (852) 23581485, Email: eekmlau@ust.hk

I. In-plane InP membranes

II. TEM analysis of epitaxial InP

III. PL spectra of InP sub-micron wires

IV. References

I. In-plane InP membranes

Figure S1a and S1b displays the top-view SEM images of the growth pattern with 5 μm and 10 μm length, respectively. Figure S1c displays the 7 μm wide oxide trench for the InP growth. The oxide trench is enclosed by sloped {111} Si facet, the top and the buried oxide layer. The anisotropy etched {111} Si facet by KOH solution is atomically flat and labeled by the white arrow. The thickness of the trench near the oxide opening is slightly larger than that close to Si, resulting from the slight etch of the oxide layer during the Si undercut process. This slightly enlarged thickness near the opening is also manifested in the gradual color change in microscope images of Figure 2a-2d, and in the TEM image of Figure 3b. The thickness difference could be reduced by further lowering the KOH concentration and temperature to increase the etch selectivity between Si and SiO_2, or adopting the soft dry etching process[1]. Figure S1d presents the global-view SEM image after the selective growth of InP, highlighting the in-plane geometry of the InP membranes and the intimate positioning with Si.

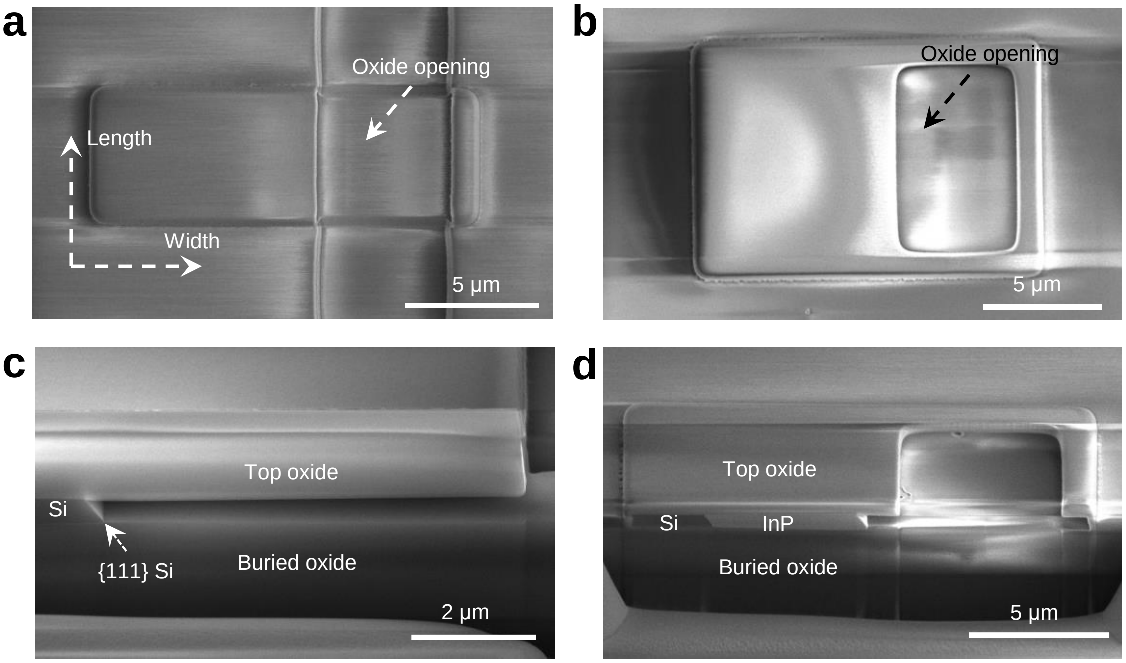

Figure S1. In-plane InP membranes. a,**b**, Top-view SEM image of growth pattern with 5 μm and 10 μm length, respectively. **c**, 52° tilted SEM image of the lateral oxide trench. **d**, Global-view 52° tilted SEM image of the in-plane InP membranes.

Figure S2a and S2b illustrate the InP membranes with pattern length of 20 μm and 50 μm, respectively. Continuous InP filled up the growth pattern, similar to the InP membranes with 5 μm and 10 μm pattern length. Although around 5 μm epitaxial width is shown here, the InP width could be readily extended to 7 μm by increasing the growth time. Along the straight epitaxial InP, occasional pits were observed as indicated in the microscope image of Figure S2b. It stems from imperfections on the InP nucleation layer, and subsequent coalescence of two separated InP nucleation. Further optimization of epitaxial parameters could improve the growth uniformity. On the other hand, it is worth mentioning that although we used oxide trench with 7 μm width here for high growth selectivity, the potential of wider trench could also be explored. In addition, performing regrowth could potentially solve this issue.

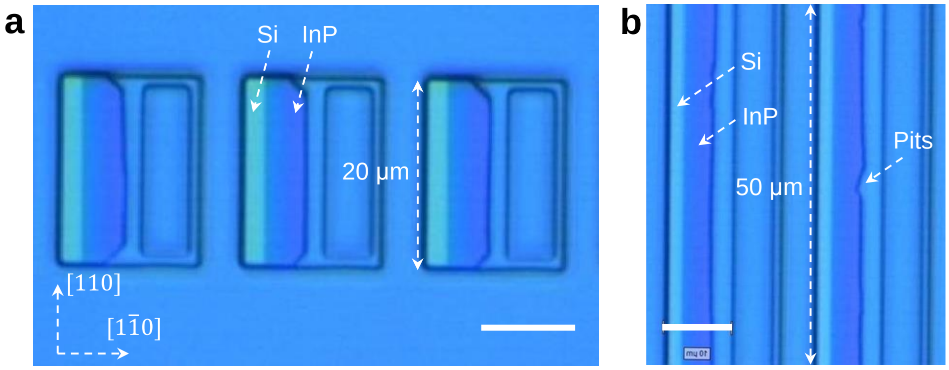

Figure S2. a,**b**, Optical microscope image of InP membranes with 20 µm and 50 µm pattern length, respectively. Scale bar, 10 µm.

II. TEM analysis of epitaxial InP

Figure S3 schematically illustrates the confinement of crystalline defects enabled by the "lateral aspect ratio trapping" approach. The growth is initiated on an etched {111} Si surface and laterally evolved along the oxide trench. Firstly, nucleating III-V from {111} Si facets often results in a high density of planar defects (stacking faults or twins) parallel to the Si surface, to efficiently relieve the misfit strains[2-4]. As indicated by red dotted lines on the sloped Si surface in Figure S3, these planar defects on the {111} Si surface are blocked by the top and buried oxide layers. Secondly, the threading dislocations (TDs) generated at the InP/Si surface propagate along the {111} glide plane and would also be trapped by the top oxide layer[5-6].

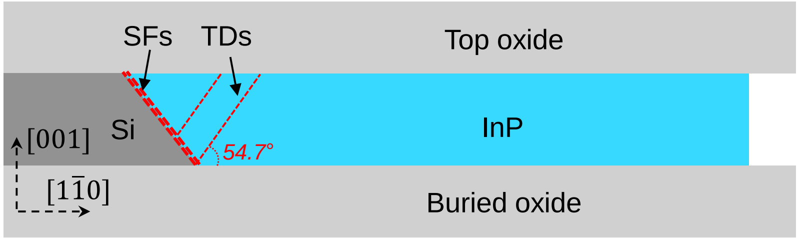

Figure S3. Defect confinement of InP laterally grown on SOI. The red dotted lines indicate the blocking of defects propagating along crystallographic orientations.

Figure S4 schematically illustrates the generation and propagation of stacking fault (SF) in the lateral growth of InP. While the ($\bar{1}\bar{1}1$) SFs could be completely trapped by the top oxide layer, the ($1\bar{1}1$)/($\bar{1}11$) SFs perpendicular to the trench direction could not be blocked and are observed in the TEM images in Figure 3h-3j. This type of SFs is also observed in the selective growth of InP[3,7], and in other zinc-blende III-V crystals (GaAs, GaSb or InAs) selectively grown on Si[8-10]. This kind of SFs terminates at the oxide surface, and therefore avoid introducing partial dislocations inside the epitaxial InP[7]. As a result, these SFs are benign to device performance[5]. Reliable InP distributed feedback (DFB) lasers, O-band and C-band telecom lasers have been demonstrated using the selectively grown III-V on Si, proving the not-compromised optoelectronic qualities[3,11-13]. Further growth optimization and clean growth surface could also reduce (and potentially eliminate) the density of this type of SFs.

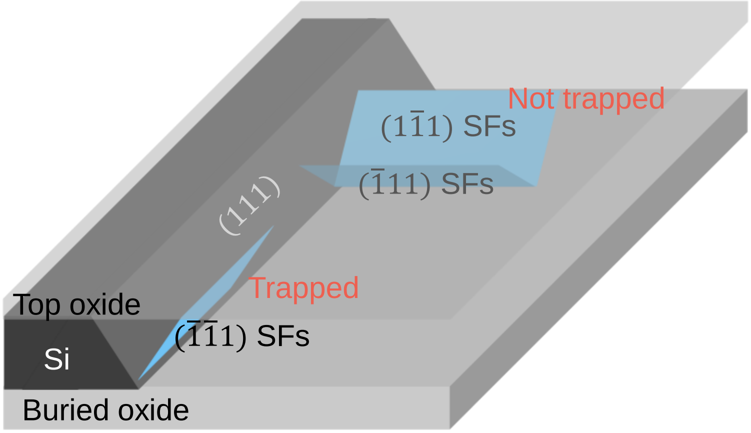

Figure S4. Schematic illustrating the SFs direction in the selective growth of InP.

III. PL spectra of InP sub-micron wires

When the pattern length was limited to around 1 µm for the growth of InP sub-micron wires, we detected the existence of PL peak located at 880 nm as shown in Figure S5. As this higher energy side PL peak was only observed in some InP sub-micron wires and not shown in membranes, we believe it was induced by the small dimension of the growth patterns. The origin of the 880 nm peak could be the existence of wurtzite-like InP phase in the sub-micron wires. In III-V nanowires with only tens of nanometer diameter, the phase mixture was more often to see[1,14-15]. Similar blue-shifted PL peak was reported in selectively grown InP inside Si trenches[3] but with less-pronounced intensity; it was also considered to be related to wurtzite-like InP phase by twins. In our case, the 880 nm peak was occasionally detected at sub-micron wire tip, but the 925 nm band PL peak was always characterized at those wire base. It indicates possible phase mixture happened during the growth process of some sub-micron wires. As enlarging InP width by increasing the growth time would normally just follow the existed crystal structure (like InP membranes), we consider that for some sub-micron wires, the surface imperfections of oxide

sidewall (by dry etching) and the confined small-scale epitaxial dimension induced the crystal impurities in the sub-micron wires.

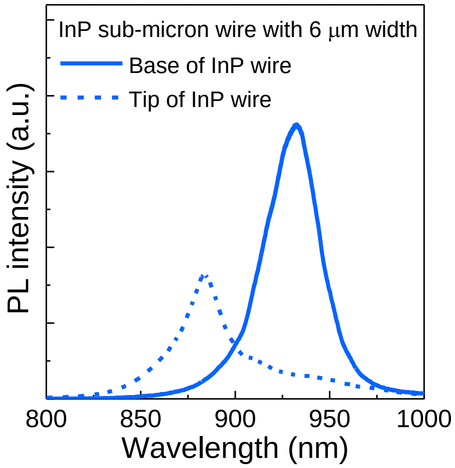

Figure S5. PL observation of an InP sub-micron wire with 500 nm pattern length and 6 μm epitaxial width.

IV. References:

1 Schmid, H. *et al*. Template-assisted selective epitaxy of III–V nanoscale devices for co-planar heterogeneous integration with Si. *Applied Physics Letters* **106**, 233101 (2015).

2 Paladugu, M. *et al*. Site selective integration of III−V materials on Si for nanoscale logic and photonic devices. *Crystal Growth & Design* **12**, 4696−4702 (2012).

3 Wang, Z. C. *et al*. Room-temperature InP distributed feedback laser array directly grown on silicon. *Nature Photonics* **9**, 837-842 (2015).

4 Li Q. *et al*. Growing antiphase-domain-free GaAs thin films out of highly ordered planar nanowire arrays on exact (001) silicon. *Applied Physics Letters* **106**, 072105 (2015).

5 Kunert, B. *et al*. How to control defect formation in monolithic III/V hetero-epitaxy on (100) Si? A critical review on current approaches. *Semiconductor Science and Technology* **33**, 093002 (2018).

6 Li Q. & Lau K. M. Epitaxial growth of highly mismatched III-V materials on (001) silicon for electronics and optoelectronics. Progress in Crystal Growth and Characterization of Materials, **63**, 4, (2017).

7 Han, Y. *et al*. InGaAs/InP quantum wires grown on silicon with adjustable emission wavelength at telecom bands. *Nanotechnology* **29** 225601 (2018).

8 Kunert, B. *et al*. III/V nano ridge structures for optical applications on patterned 300 mm silicon substrate. *Applied Physics Letters* **109**, 091101 (2016).

9 Li, Q., Lai, B. & Lau, K. M. Epitaxial growth of GaSb on V-grooved Si (001) substrates with an ultrathin GaAs stress relaxing layer. *Applied Physics Letters* **111**, 172103 (2017).

10 Yan, Z., Han, Y. & Lau, K. M. InAs nano-ridges and thin films grown on (001) silicon substrates. *Journal of Applied Physics* **128**, 035302 (2020).

11 Han, Y. *et al*. Bufferless 1.5 µm III-V lasers grown on Si-photonics 220 nm silicon-on-insulator platforms. *Optica* **7**, 148-153 (2020).

12 Shi, Y. T. *et al*. Optical pumped InGaAs/GaAs nano-ridge laser epitaxially grown on a standard 300-mm Si wafer. *Optica* **4**, 1468-1473 (2017).

13 Han, Y. *et al*. Telecom InP/InGaAs nanolaser array directly grown on (001) silicon-on-insulator. *Optics Letters* **14**, 4 (2019).

14 Mauthe, S. *et al*. High-speed III-V nanowire photodetector monolithically integrated on Si. *Nature Communications* **11**, 4565 (2020).

15 Borg, M. *et al*. High-Mobility GaSb Nanostructures Cointegrated with InAs on Si. *ACS Nano*, **11**, 2554−2560 (2017).
